# Role of Metabolic Genes in Blood Arsenic Concentrations of Jamaican Children with and without Autism Spectrum Disorder

**DOI:** 10.3390/ijerph110807874

**Published:** 2014-08-06

**Authors:** Mohammad H. Rahbar, Maureen Samms-Vaughan, Jianzhong Ma, Jan Bressler, Katherine A. Loveland, Manouchehr Ardjomand-Hessabi, Aisha S. Dickerson, Megan L. Grove, Sydonnie Shakespeare-Pellington, Compton Beecher, Wayne McLaughlin, Eric Boerwinkle

**Affiliations:** 1 Division of Epidemiology, Human Genetics, and Environmental Sciences (EHGES), University of Texas School of Public Health at Houston, Houston, TX 77030, USA; E-Mail: Eric.Boerwinkle@uth.tmc.edu; 2 Division of Clinical and Translational Sciences, Department of Internal Medicine, Medical School, University of Texas Health Science Center at Houston, Houston, TX 77030, USA; E-Mail: Jianzhong.Ma@uth.tmc.edu; 3 Biostatistics/Epidemiology/Research Design (BERD) Component, Center for Clinical and Translational Sciences (CCTS), University of Texas Health Science Center at Houston, Houston, Texas 77030, USA; E-Mails: Manouchehr.A.Hessabi@uth.tmc.edu (M.A.-H.); Aisha.S.Dickerson@uth.tmc.edu (A.S.D.); 4 Department of Child & Adolescent Health, The University of the West Indies (UWI), Mona Campus, Kingston 7, Jamaica; E-Mails: msammsvaughan@gmail.com (M.S.-V.); sydonniesp@gmail.com (S.S.-P.); 5 Human Genetics Center, University of Texas School of Public Health at Houston, Houston, TX 77030, USA; E-Mails: Jan.Bressler@uth.tmc.edu (J.B.); Megan.L.Grove@uth.tmc.edu (M.L.G.); 6 Department of Psychiatry and Behavioral Sciences, University of Texas Medical School at Houston, Houston, TX 77054, USA; E-Mail: Katherine.A.Loveland@uth.tmc.edu; 7 Department of Basic Medical Sciences, The University of the West Indies, Mona Campus, Kingston 7, Jamaica; E-Mails: compton.beecher@uwimona.edu.jm (C.B.); wayne.mclaughlin@uwimona.edu.jm (W.M.); 8 Caribbean Genetics (CARIGEN), The University of the West Indies, Mona Campus, Kingston 7, Jamaica

**Keywords:** arsenic, autism spectrum disorder (ASD), glutathione S-transferase (*GST*) genes, detoxification, interactions

## Abstract

Arsenic is a toxic metalloid with known adverse effects on human health. Glutathione-S-transferase (GST) genes, including *GSTT1*, *GSTP1*, and *GSTM1*, play a major role in detoxification and metabolism of xenobiotics. We investigated the association between GST genotypes and whole blood arsenic concentrations (BASC) in Jamaican children with and without autism spectrum disorder (ASD). We used data from 100 ASD cases and their 1:1 age- and sex-matched typically developing (TD) controls (age 2–8 years) from Jamaica. Using log-transformed BASC as the dependent variable in a General Linear Model, we observed a significant interaction between *GSTP1* and ASD case status while controlling for several confounding variables. However, for *GSTT1* and *GSTM1* we did not observe any significant associations with BASC. Our findings indicate that TD children who had the Ile/Ile or Ile/Val genotype for *GSTP1* had a significantly higher geometric mean BASC than those with genotype Val/Val (3.67 µg/L *vs.* 2.69 µg/L, *p* < 0.01). Although, among the ASD cases, this difference was not statistically significant, the direction of the observed difference was consistent with that of the TD control children. These findings suggest a possible role of *GSTP1* in the detoxification of arsenic.

## 1. Introduction

Arsenic is a widespread environmental toxicant with harmful effects on human health. In some developing countries, arsenic exposure through drinking water and food is a serious threat to human health [1,2]. Inorganic arsenic is an established carcinogen [3] that affects neurodevelopment [4] and may also play a role in cardiovascular disease [5,6,7] and diabetes [8,9,10,11]. Arsenic exposure is also associated with other diseases such as pigmented skin lesions, blackfoot disease (gangrene), and hypertension [12]. In addition, there is increasing evidence that arsenic exposure is associated with neurodevelopmental disorders in infants and children [13,14]. Organic arsenic, such as arsenobetaine and arsenocholine, are much less toxic than inorganic arsenic [15]; however, some studies have shown that organic arsenic can also have adverse effects on neurodevelopment [4]. Several studies have investigated the possible association between exposure to arsenic and autism spectrum disorder (ASD), but their findings are conflicting [16,17,18,19,20,21,22,23,24]. For example, some case-control studies have shown that children with ASD had higher mean hair or plasma arsenic levels than typically developing (TD) children [18,19,20,21], although other studies have shown an opposite association [17,24]. On the other hand, other studies have shown no associations between arsenic exposure and ASD [16,22].

Genetic regulatory mechanisms are involved in detoxification of heavy metals including arsenic [25]. However, the mechanism of human arsenic metabolism and the relationship to the related disease susceptibility is not fully understood [26]. In humans, inorganic arsenic ingested through drinking water and contaminated food is metabolized to dimethyl arsenic [27], and there is a consensus in animal and human studies that chronic exposure to arsenic causes oxidative stress through generation of reactive oxygen species [27,28,29,30,31]. Recent reports have identified oxidative stress as a possible factor associated with arsenic carcinogenesis [32]. Although a classical scheme consists of a series of reductions and oxidations coupled with methylations [33,34], it was recently proposed that the reductive methylation process interacts with binding proteins [35,36]. Glutathione S-transferase (GST) genes encode enzymes that are associated with detoxification of xenobiotics by catalyzing the conjugation with reduced glutathione [37,38]. The GST superfamily includes seven genes: alpha, mu, omega, pi, theta, sigma, and zeta [38]. Two genes play an important role in the arsenic biotransformation process: arsenic methyltransferase (AS3MT) and glutathione S-transferase omega (*GSTO*) [39]. It has been shown that *GSTO1* and *GSTO2* are involved in the reduction activities of arsenate, monomethylarsonic acid (MMeAsIII), and dimenthylarisinic acid (DMeAsIII) [27].

It is well-established that glutathione S-transferase pi (*GSTP1*) and glutathione S-transferase mu (*GSTM1*) also play important roles in detoxification of xenobiotics [40]. Polymorphisms in these GST genes may have effects on the behavior of these enzymes involved with the maintenance of cellular glutathione (GSH) level. Studies have shown that polymorphisms in GST genes, particularly *GSTM1*, may be associated with increased susceptibility to mercury toxicity [25,41] ethyl mercury sensitization [42] and xenobiotic toxicity, including polychlorinated biphenyl (PCBs) [43] and polycyclic aromatic hydrocarbons [44]. On the other hand, polymorphisms in the GST genes, which are responsible for the production of GSH, have been associated with ASD [45,46,47], and deficiencies in GSH have been linked to ASD [45,48,49,50]. Therefore, it is highly relevant to examine potential interactions between GST genes and ASD in relation to detoxification of heavy metals, such as arsenic.

Based on data from 65 pairs of sex- and age-matched ASD cases and TD controls from our Jamaican Autism Study, we have previously reported a lack of association between blood arsenic concentrations and ASD in a multivariable model that adjusted for potential confounding factors such as source of drinking water, age of mother at child’s birth, parental education levels, socioeconomic status, and consumption of some fruits, vegetables, and seafood [22]. In addition, we reported that blood arsenic concentrations in Jamaican children are about four times higher than those of children in the US or Canada [22]. Since Jamaican soils have high arsenic levels [51], and Jamaicans consume large amounts of fruits and vegetables that contain arsenic [52], as well as seafood that has been associated with higher blood arsenic concentrations in young children [22], we believe the Jamaican community has had continuous exposure to arsenic. In the present research, we investigated the role of three GST genes (*GSTP1*, *GSTM1*, and *GSTT1*) in relation to blood arsenic concentrations in Jamaican children with and without ASD.

## 2. Materials and Methods

### 2.1. General Description

The Jamaican Autism Study is an age- and sex-matched case-control study of Jamaican children between 2–8 years of age that were enrolled during December 2009–May 2012. The overall goal of the Jamaican Autism Study is to investigate whether environmental exposures to arsenic, mercury, lead, cadmium, and manganese play a role in ASD and to assess the role of three glutathione S-transferase genes (*GSTT1*, *GSTM1*, and *GSTP1*) and their interactions with these heavy metals in relation to ASD. Detailed information regarding the recruitment and assessment of ASD cases and TD controls was reported earlier [22,53,54]. In brief, children in the University of the West Indies’ (UWI) Jamaica Autism Database who were previously identified as being at risk for an ASD based on Diagnostic Statistical Manual of Mental Disorders (DSM-IV-TR) criteria [55] and the Childhood Autism Rating Scale (CARS) [56], were invited to participate for reassessment of their ASD status for this study. We administered the Autism Diagnostic Observation Schedule (ADOS) [57] and the Autism Diagnostic Interview-Revised (ADI-R) [58] to confirm the diagnosis of ASD. For ascertainment of ASD status, we used standard algorithms developed for scoring ADOS and ADI-R and established cut-off points. Each ASD case was confirmed based on both ADI-R and all three domains in ADOS. For each confirmed ASD case, an age- and sex-matched control was identified from schools and well-child clinics. The criteria for matching required that the age of the control child be within six months of the matched ASD case. We administered the Lifetime form of the Social Communication Questionnaire (SCQ) [59] to the parents/guardians of potential control children to rule out symptoms of ASD.

We also administered a pre-tested questionnaire to the parents/guardians of both ASD cases and TD controls to collect their demographic and socioeconomic status (SES) information, such as ownership of a car by the family, parental education levels, and potential exposure to heavy metals through drinking water sources and food, especially the types and frequency of fruits, vegetables, and seafood consumed by the children. The types of seafood considered included: (1) saltwater fish; (2) freshwater fish (pond fish, tilapia); (3) sardine or mackerel (canned fish); (4) tuna (canned fish); (5) salted fish (pickled mackerel); (6) shellfish (lobster, crabs); and (7) shrimp. The types of fruits and vegetables were classified into different categories, based on their characteristics and species. Details regarding the categories of fruits and vegetables were reported earlier [22].

About 5 mL of venous whole blood was drawn from each child. Samples for trace metals analysis (2 mL) were stored at −20 °C degrees while remaining samples for genetic analysis (3 mL) were stored at −80 °C degrees at UWI in Kingston, Jamaica. In addition, 2 mL of saliva was also collected from each child. Whole blood samples for trace metal analysis were shipped to the Michigan Department of Community Health (MDCH), and the whole blood and saliva samples for genetic analysis were shipped to the University of Texas School of Public Health at Houston. All parents/guardians provided written informed consent. This study was approved by the Institutional Review Boards of the University of Texas Health Science Center at Houston (UTHealth) and UWI, Mona campus, in Kingston, Jamaica. The data presented herein represent analysis of 100 1:1 matched case-control pairs for whom we had complete data.

### 2.2. Assessment of Arsenic Exposures

Arsenic exposure can be assessed using various biological specimens including blood, hair, urine, and fingernails depending on duration of exposure. Arsenic levels in the hair or fingernails reflect exposure from the past several months [60,61]. For measuring recent arsenic exposure (several days), most literature recommends assessment of arsenic in urine (adjusted for creatinine in urine) as a more reliable method [4]. For example, a study in Bangladesh showed high correlation between total blood arsenic concentrations and creatinine-adjusted urine arsenic concentrations (r = 0.85) [62]. Also, arsenic in blood has been found to be reliable for evaluation of exposure in populations that are continuously exposed to arsenic from different sources including drinking water and food. Since the Jamaican community has continuous exposure to arsenic through various sources including fruits and vegetables [52], as well as seafood [22], we believe arsenic in whole blood should be considered as a reliable biomarker for arsenic exposure. In this study, total blood arsenic concentrations were assessed by the Trace Metals Lab at MDCH, which is certified by the Centers for Disease Control and Prevention (CDC). MDCH followed a fully validated protocol for analyzing arsenic in blood samples with a detection limit of 1.0 µg/L. All samples were diluted and analyzed on a PerkinElmer Elan DRC II inductively-coupled plasma mass spectrometer (PerkinElmer, Waltham, MA, USA).

### 2.3. Genetic Analysis

Whole blood was collected in EDTA lavender top vacutainers for each participant at UWI. Blood components were centrifuged and separated into plasma, buffy coat, and red blood cell aliquots, then stored at −80 °C for future use. Frozen specimens were sent to the UTHealth School of Public Health, Human Genetics Center using the CryoPort high volume liquid nitrogen dry vapor shipper (CryoPort, Inc.; San Diego, CA, USA). Saliva was collected with Oragene Discover DNA Collection Kits for Research (OGR-500; DNA Genotek, Inc.; Kanata, ON, Canada). If the child had difficulty in spitting 2 mL of saliva, then the Oragene Discover OGR-575 for Assisted Collection with sponges was used (DNA Genotek). Saliva samples were shipped from UWI to UTHealth at ambient temperature.

Genomic DNA was isolated from buffy coat using the Gentra PUREGENE Blood Kit (Qiagen, N.V.; Venlo, The Netherlands) in accordance with the manufacturer’s protocol. If a buffy coat was not available, then DNA was isolated from the saliva sample with the Gentra PUREGENE DNA Purification Kit (Qiagen protocol 400244 Rev A) according to the methods established by DNA Genotek [63]. The *GSTT1* and *GSTM1* genotypes were detected using a multiplex polymerase chain reaction (PCR) reaction and methods established by Li *et al.* (2000) [64]. In this assay, the absence of a 130 bp product indicates that the individual is homozygous null for *GSTT1*. Similarly, the absence of a 230 bp product indicates homozygous deficiency for the *GSTM1* gene. As a quality control, a beta-globin amplicon of 280 bp was included in the assay. The *GSTP1* polymorphism Ile105Val (rs1695) was genotyped using the TaqMan Drug Metabolism SNP Genotyping Assay C_3237198_20 using the thermal cycling parameters recommended by Life Technologies (Grand Island, NY, USA). The ABI 7700 Sequence Detection System (Life Technologies) was used for ascertainment of the genotypes.

### 2.4. Statistical Analysis

Descriptive analyses were conducted to compare demographic and SES characteristics of ASD cases and TD controls. As the distribution of blood arsenic concentrations is skewed, the data were transformed using the natural logarithm (ln) to produce an approximately normal distribution. The means of the log transformed blood arsenic concentrations were transformed back to their original scale (*i.e.*, µg/L) by applying the natural exponential function, herein called geometric means.

For the *GSTM1* and *GSTT1* genes, since the assay does not distinguish between a normal homozygote (I/I) and a heterozygote (I/D), we considered only a recessive model for both of them using a binary variable to represent their genotype: I* and DD. For the *GSTP1* gene, all three genotypes, Ile/Ile, Ile/Val and Val/Val, were available. The effect of *GSTP1* is still controversial, not only for the genetic models but also is for the direction of influence. In the literature, different genetic models (e.g., dominant or recessive models) have been proposed for the *GSTP1* polymorphism and the effect of a genotype (e.g., Val/Val) may be protective for some diseases and deleterious for others. For example, a recessive model of *GSTP1* was used in a previous study [65], where a significant association was found between low-stage prostate cancer and the Val/Val genotype in a meta-analysis. On the other hand, a dominant model was considered by Chen *et al.* (2010) and significant association of Ile/Val or Val/Val genotype with hepatocellular carcinoma was observed [66]. A study by Aynacioglu (2004) reported that Val/Val homozygotes appear to be somewhat protective from developing asthma compared to individuals with the other two genotypes [67]. In this study, we therefore analyzed the *GSTP1* gene using different genetic models, including the recessive (Ile/* *vs.* Val/Val) and the full models. For the *GSTP1* polymorphism, we tested the Hardy-Weinberg equilibrium using the Chi-square test in the TD control group.

In this study, we used conditional logistic regression (CLR) models to assess the association between ASD status and various exposure variables including genotypes for *GSTP1*, *GSTM1*, and *GSTT1.* Subsequently, we used General Linear Models (GLMs) with the log-transformed blood arsenic concentrations as the dependent variable to investigate the role of the three aforementioned GST genes as well as other exposure variables in blood arsenic concentrations. In order to minimize any potential effects of multicollinearity due to a high correlation between maternal and paternal education levels, we created a binary variable indicating whether both parents had education up to high school or at least one of the parents obtained education beyond high school. In all GLMs, we also controlled for the clustering effect of matching by including an appropriate number of dummy variables that represented the matched pairs (e.g., 99 dummy variables for 100 matched pairs). In multivariable GLMs, we assessed interactions between each of the three GST genes and ASD case status while controlling for potential confounding variables that included SES (car ownership), maternal age at child’s birth, parental education levels, source of drinking water, consumption of “yam, sweet potato, or dasheen”, “carrot or pumpkin”, “callaloo, broccoli, or pak choi”, “cabbage”, “avocado”, and frequency of seafood consumption. Since determination of potential confounding variables in models that involve interactive effects does not follow the classical definition of confounders in additive models, we adjusted for potential confounders that were determined *a priori* from our earlier work [22]. Since we found a significant interaction between ASD status and *GSTP1* genotypes in relation to blood arsenic concentrations, we used the CONTRAST statement in PROC GLM in SAS [68] to test whether there is a significant difference in geometric mean blood arsenic concentrations between various genotypes of *GSTP1*, separately for ASD cases and TD controls. We calculated unadjusted and adjusted geometric mean blood arsenic concentrations for groups of children with different GST genotypes (e.g., *GSTP1*). Though not the main objective of this paper, we used univariable and multivariable GLMs to compare geometric mean blood arsenic concentrations by ASD status based on the 100 matched pairs to our previously reported findings based on 65 matched pairs [22]. For details, see supplementary material.

## 3. Results

The mean age of children with ASD was 68.4 months and the mean age for TD children in the control group was 69.3 months. About 85.0% of the ASD cases and TD controls were male. Nearly all of the ASD cases (93.0%) and TD controls (99.0%) were Afro-Caribbean. Similarly, 93.0% of mothers and 99.0% of fathers were Afro-Caribbean. Our data indicate a prevalence of 26.0% and 22.0% for the *GSTM1* and *GSTT1* null genotype, respectively, for TD children. There was no significant deviation from Hardy-Weinberg equilibrium for the *GSTP1* polymorphism in the TD controls (*p* = 0.69). In addition, there were no significant differences between ASD cases and TD controls with respect to the genotype frequencies of *GSTM1*, *GSTP1*, and *GSTT1*, (all *p* > 0.21). Demographic and other characteristics of children and their parents by ASD case status are presented in Table 1.

A comparison of dietary consumption between ASD cases and TD controls revealed that a significantly lower proportion of ASD cases reported eating sardine or mackerel (Matched Odds Ratio (MOR) = 0.26, 95% CI (0.11, 0.64), *p* < 0.01) and cabbage (MOR = 0.15, 95% CI (0.06, 0.38), *p* < 0.01). In addition, a significantly lower proportion of ASD cases reported fruit and vegetable consumption. Comparisons of other variables related to dietary consumption between children with and without ASD are displayed in Table 2.

A comparison of geometric mean blood arsenic concentrations between children who had different levels and types of exposures revealed the following findings. In the univariable analysis, there was no significant association between blood arsenic concentrations with the three GST genes (*GSTM1* (*p* = 0.95), *GSTT1* (*P* = 0.28), and *GSTP1* genotypes (all *p >* 0.11)). Furthermore, children who ate salt water fish had a significantly higher geometric mean blood arsenic concentration than those who did not eat this type of fish (3.69 µg/L *vs.* 3.11 µg/L; *p* = 0.03). In addition, a significant difference was found between geometric mean blood arsenic concentrations of children whose family consumed seafood more than 6 meals per week and those who did not (3.97 μg/L *vs.* 3.39 μg/L, respectively, *p* = 0.01). Associations of other exposure variables with blood arsenic concentrations are reported in Table 3.

We also examined the difference between ASD cases and TD control groups with respect to arsenic blood concentrations based on data from 100 matched pairs and compared these results with our preliminary analysis based on 65 matched pairs [22]. Although the unadjusted findings from univariable analyses are different as shown in Table 4, the findings from multivariable analyses are consistent, indicating a lack of significant association between blood arsenic concentrations and ASD status based on additive models [22]. The data for such comparisons are displayed in Table 4.

**Table 1:** Characteristics of children and their parents by autism spectrum disorder (ASD) case status (100 matched pairs).

Variables	Categories	ASD Case (*n* = 100) N (%)	TD Control (*n* = 100) N (%)	*p*-value *
**Child’s sex**	Male	85 (85.0)	85 (85.0)	1.00
**Child’s age** (months)	Age < 48	16 (16.0)	13 (13.0)	0.64
	48 ≤ age < 72	46 (46.0)	47 (47.0)	
	Age ≥ 72	38 (38.0)	40 (40.0)	
**Child’s race**	Afro-Caribbean	93 (93.0)	99 (99.0)	0.25
**Maternal age *^a^*** (at child’s birth)	<35 years	76 (76.0)	84 (88.4)	0.02
	≥35 years	24 (24.0)	11 (11.6)	
**Paternal age *^b^*** (at child’s birth)	<35 years	47 (48.5)	66 (71.7)	<0.01
	≥35 years	50 (51.5)	26 (28.3)	
**Maternal race**	Afro-Caribbean	93 (93.0)	99 (99.0)	0.25
**Paternal race *^c^***	Afro-Caribbean	94 (94.9)	99 (99.0)	0.67
**Maternal education *^d^*** (at child’s birth)	Up to high school ^†^	53 (53.0)	75 (76.5)	<0.01
	Beyond high school ^††^	47 (47.0)	23 (23.5)	
**Paternal education *^e^*** (at child’s birth)	Up to high school ^†^	53 (54.6)	85 (87.6)	<0.01
	Beyond high school ^††^	44 (45.4)	12 (12.4)	
**Socioeconomic status (SES)**	Car ownership	68 (68.0)	37 (37.0)	<0.01
*GSTP1*	Ile/Ile	30 (30.0)	25 (25.0)	0.56
	Ile/Val	52 (52.0)	52 (52.0)	
	Val/Val	18 (18.0)	23 (23.0)	
*GSTM1*	DD *^f^*	27 (27.0)	26 (26.0)	0.87
	I/I or I/D *^g^*	73 (73.0)	74 (74.0)	
*GSTT1*	DD *^f^*	30 (30.0)	22 (22.0)	0.21
	I/I or I/D *^g^*	70 (70.0)	78 (78.0)	

* *p*-values are based on Wald’s test in conditional logistic regression models that compares the distribution of independent variables between and ASD case and TD control groups; ^†^ Up to high school education means attended Primary/Jr. Secondary, and Secondary/High/Technical schools;^††^ Beyond high school education means attended a Vocational, Tertiary College, or University; ^a^ Maternal age was missing for 5 TD controls; ^b^ Paternal age were missing for 3 ASD cases and 8 TD controls; ^c^ Paternal race was missing for 1 ASD case; ^d^ Maternal education was missing for 2 controls; ^e^ Paternal education was missing for 3 cases and 3 controls; ^f^ DD indicates the null alleles for *GSTT1* and *GSTM1*; ^g^ I/I or I/D indicate the homozygote (I/I) or a heterozygote (I/D) for *GSTT1* and *GSTM1*.

In multivariable analyses, we detected a significant interaction between ASD status and *GSTP1* genotype in relation to blood arsenic concentrations while controlling for the aforementioned potential confounders. Specifically, in a full genetic model we found a significant interaction between ASD status and *GSTP1* genotypes in relation to blood arsenic concentrations (*p* = 0.04). As shown in Table 5, based on the full genetic model, TD children with genotype Ile/Val had significantly higher adjusted geometric mean blood arsenic concentration than those with genotype Val/Val (3.80 µg/L *vs.* 2.72 µg/L, *p* < 0.01). Similarly, in the TD controls, children with genotype Ile/Ile had significantly higher adjusted geometric mean blood arsenic concentration than those with genotype Val/Val (3.72 µg/L *vs.* 2.72 µg/L, *p* = 0.02). Additionally, in the recessive model, TD children with Ile/Ile or Ile/Val genotypes had significantly higher adjusted geometric mean blood arsenic concentrations compared to those with the Val/Val genotype (3.67 µg/L *vs.* 2.69 µg/L, *p* < 0.01).

**Table 2:** Associations between dietary consumption and ASD case status using Conditional Logistic Regression (CLR) (200 children or 100 matched pairs).

Exposure variables	Category		ASD Case N (%)	TD Control N (%)	Matched OR (MOR)	95% CI for MOR	*p*-value *^d^*
**Source of** **drinking water** ***^a^***	Piped water		94 (94.0)	95 (96.0)	0.67	(0.19, 2.36)	0.53
**Source of water** **for cooking** ***^b^***	Piped water		94 (94.0)	95 (96.0)	0.67	(0.19, 2.36)	0.53
**Fruits and vegetables consumption *^c^***	Root vegetables	A. Yam, sweet potato, or dasheen	73 (73.0)	82 (82.8)	0.52	(0.25, 1.07)	0.08
		B. Carrot or pumpkin	86 (86.0)	98 (99.0)	0.08	(0.01, 0.59)	0.01
	Leafy vegetables	A. Lettuce	47 (47.0)	62 (62.6)	0.57	(0.33, 0.97)	0.04
		B. Callaloo, broccoli, or pakchoi	72 (72.0)	94 (94.9)	0.18	(0.07, 0.46)	<0.01
		C. Cabbage	66 (66.0)	94 (94.9)	0.15	(0.06, 0.38)	<0.01
	Fruits	Tomatoes	62 (62.0)	85 (85.9)	0.23	(0.10, 0.51)	<0.01
		Ackee	58 (58.0)	92 (92.9)	0.06	(0.01, 0.23)	<0.01
		Avocado	29 (29.0)	68 (68.7)	0.19	(0.09, 0.38)	<0.01
		Green banana	67 (67.0)	90 (90.9)	0.27	(0.13, 0.57)	<0.01
		Fried plantains	70 (70.0)	89 (89.9)	0.17	(0.06, 0.48)	<0.01
**Seafood consumption**	Ate salt water fish		77 (77.0)	89 (89.0)	0.40	(0.18, 0.91)	0.03
	Ate fresh water fish (Pond fish, Tilapia)		46 (46.0)	52 (52.0)	0.75	(0.41, 1.38)	0.36
	Ate sardine, mackerel (Canned fish)		75 (75.0)	92 (92.0)	0.26	(0.11, 0.64)	<0.01
	Ate tuna (Canned fish)		31 (31.0)	44 (44.0)	0.55	(0.30, 1.02)	0.06
	Ate salted fish (Pickled mackerel)		70 (70.0)	93 (93.0)	0.15	(0.05, 0.42)	<0.01
	Ate shellfish (Lobsters, Crabs)		7 (7.0)	14 (14.0)	0.42	(0.15, 1.18)	0.10
	Ate shrimp		19 (19.0)	27 (27.0)	0.62	(0.31, 1.24)	0.17

*^a^* Source of drinking water was missing for 1 control; *^b^* Source of water for cooking was missing for 1 control; *^c^* For all variables under fruits and vegetables consumption data was missing for 1 control; *^d^* *p*-values are based on Wald’s test in conditional logistic regression models that compares the distribution of dietary consumption between and ASD case and TD control groups.

**Table 3:** Associations of various exposure variables with blood arsenic concentrations based on univariable General Linear Models (100 matched pairs).

Exposure variables	Category		Yes		No		*p*-value *^g^*
			Mean As * (μg/L)	N	Mean As * (μg/L)	N	
**Socioeconomic status **	Own a car		3.64	105	3.52	95	0.58
**Maternal age** *^a^* (at child’s birth)	≥35 years		3.71	35	3.54	160	0.56
**Parental education levels** *^b^* (at child’s birth)	At least one of the parents had education beyond high school		3.57	94	3.61	98	0.84
**Source of drinking water** *^c^*	Piped water		3.52	189	4.83	10	0.02
**Fruits and vegetables consumption** *^d^*	Root vegetables	A. Yam, sweet potato, or dasheen	3.73	155	3.09	44	0.01
		B. Carrot or pumpkin	3.61	184	3.28	15	0.41
	Leafy vegetables	A. Lettuce	3.56	109	3.61	90	0.83
		B. Callaloo, broccoli, or pak choi	3.61	166	3.42	33	0.47
		C. Cabbage	3.66	160	3.27	39	0.14
	Legumes	String beans	3.61	75	3.57	124	0.84
	Fruits	Tomatoes	3.61	147	3.49	52	0.65
		Ackee	3.68	150	3.31	49	0.19
		Avocado	3.86	97	3.34	102	0.02
**Seafood consumption**	High seafood consumption (more than 6 meals per week)		3.97	71	3.39	129	0.01
	Frequency of seafood meals consumed weekly		NA	-	NA	-	0.06
	Ate salt water fish		3.69	166	3.11	34	0.03
	Ate fresh water fish (pond fish, tilapia)		3.57	98	3.60	102	0.88
	Ate sardine, mackerel (canned fish)		3.64	167	3.30	33	0.22
	Ate tuna (canned fish)		3.83	75	3.44	125	0.09
	Ate salted fish (pickled mackerel)		3.70	163	3.12	37	0.03
	Ate shellfish (lobsters, crabs)		3.71	21	3.57	179	0.71
	Ate shrimp		3.42	46	3.64	154	0.39
**Genes**	*GSTT1* (I*) *^e^*		3.52	148	3.78	52	0.28
	*GSTM1* (I*) *^e^*		3.59	147	3.57	53	0.95
	*GSTP1* (Ile/Ile) *^f^*		3.67	55	3.55	145	0.64
	*GSTP1* (Val/Val) *^f^*		3.26	41	3.67	159	0.11
	*GSTP1* (Ile/Val) *^f^*		3.67	104	3.49	96	0.39

NA: not applicable, because frequency of seafood meals consumed weekly is analyzed as a continuous variable; * Mean As indicates the geometric mean = Exp. (Mean (ln As)). The “Yes” column includes participants who met the category specified in front of each exposure variable; The “No” column includes participants who did not meet the category specified in front of each exposure variable; *^a^* Maternal age was missing for 5 participants; *^b^* Parental education levels was missing for 8 participants; *^c^* Source of drinking water was missing for 1 participant; *^d^* Fruits and vegetables consumption was missing for 1 participant; *^e^* I* indicates the homozygote (I/I) or a heterozygote (I/D) for *GSTT1 and* *GSTM1*; *^f^* *GSTP1* has 3 categories (Ile/Ile, Ile/Val, Val/Val); *^g^* *P*-values are based on GLMs that compare geometric mean blood arsenic concentrations between children who were exposed and those who were not exposed to the situation described in the “Yes” column (See supplementary material for detailed description of GLMs used to obtain the results in this table).

**Table 4:** Unadjusted and adjusted mean blood arsenic concentrations for ASD cases and their matched TD controls based on General Linear Models (GLM) based on 65 and 100 matched pairs.

Additive Models		ASD Cases Mean As(μg/L)	TD Controls Mean As (μg/L)	*p*-value
**65 matched pairs**	Unadjusted	4.03	4.48	<0.01
	Adjusted *^a^*	4.36	4.65	0.23
**100 matched pairs**	Unadjusted	3.49	3.68	0.20
	Adjusted *^a^*	3.57	3.46	0.64

Mean As indicates the geometric mean = Exp. (Mean (lnAs)). *p*-values are related to the comparison between ASD case and TD control groups with respect to geometric mean blood arsenic concentrations; *^a^* Factors adjusted for in the final GLM include: SES (car ownership), maternal age at child’s birth, parental education levels, source of drinking water, consumption of “yam, sweet potato, or dasheen”, “carrot or pumpkin”, “callaloo, broccoli, or pak choi”, “cabbage”, “avocado”, and frequency of seafood consumption” (See supplementary material for detailed description of GLMs used to obtain the results in this table).

**Table 5:** Unadjusted and adjusted geometric mean blood arsenic concentrations by *GSTP1* genotypes based on General Linear Models (GLM) that includes interaction between *GSTP1* and ASD case status (100 matched pairs).

Models	Gene	(Column A) Genotypes compared	Referent Genotypes	Group	Unadjusted			Adjusted *^c^*		*P ^d^*
					Mean As (μg/L) of children with genotypes in Column A *	Mean As (μg/L) of children with referent genotypes*	*P ^d^*	Mean As (μg/L) of children with genotypes in Column A *	Mean As (μg/L) of children with referent genotypes *	
**Full** *^a^*	*GSTP1*	Ile/Ile	Ile/Val	TD Control	3.82	3.90	0.83	3.72	3.80	0.84
	*GSTP1*	Ile/Ile	Ile/Val	ASD Case	3.55	3.46	0.79	4.04	3.59	0.27
	*GSTP1*	Ile/Ile	Val/Val	TD Control	3.82	3.10	0.08	3.72	2.72	0.02
	*GSTP1*	Ile/Ile	Val/Val	ASD Case	3.55	3.50	0.91	4.04	3.33	0.14
	*GSTP1*	Ile/Val	Val/Val	TD Control	3.90	3.10	0.03	3.80	2.72	<0.01
	*GSTP1*	Ile/Val	Val/Val	ASD Case	3.46	3.50	0.92	3.59	3.33	0.52
**Recessive** *^b^*	*GSTP1*REC	Ile/Ile or Ile/Val	Val/Val	TD Control	3.87	3.10	0.02	3.67	2.69	<0.01
	*GSTP1*REC	Ile/Ile or Ile/Val	Val/Val	ASD Case	3.49	3.49	0.99	3.71	3.29	0.29

* Mean As indicates the geometric mean = Exp. (Mean (ln As)). *^a^* *GSTP1* in the full model has 3 categories (Ile/Ile, Ile/Val, Val/Val); *^b^* *GSTP1* (REC) = *GSTP1* in the recessive model has two categories (Val/Val, Ile/Ile or Ile/Val); *^c^* Factors adjusted for include: SES (car ownership), maternal age at child’s birth, parental education levels, source of drinking water, consumption of “yam, sweet potato, or dasheen”, “carrot or pumpkin”, “callaloo, broccoli, or pak choi”, “cabbage”, “avocado”, and frequency of seafood consumption; *^d^ P*-values are for the comparison of mean blood arsenic concentrations of children with genotypes in “Column A” compared to those with “referent genotypes”, stratified by ASD case status, based on CONTRAST option in the SAS program for GLMs as described in the methods section (See supplementary material for detailed description of GLMs used to obtain the results in this table).

However, in the ASD case group, there were no significant associations between genotypes of *GSTP1* and blood arsenic concentrations, though the observed differences in blood arsenic concentrations had similar trends. For example, in the recessive model, ASD cases with Ile/Ile or Ile/Val genotypes had adjusted geometric mean blood arsenic concentration of 3.71 µg/L compared to 3.29 µg/L for ASD cases with the Val/Val genotype (*p* = 0.29). Similar analyses for *GSTT1* and *GSTM1* did not result in any significant associations with the blood arsenic concentrations in children with or without ASD (data based on multivariable analysis are not shown for *GSTT1* and *GSTM1*). Details regarding the *GSTP1* results are shown in Table 5.

## 4. Discussion

In this study, we have investigated the role of three GST genes (*GSTT1*, *GSTM1*, and *GSTP1*) in blood arsenic concentrations of Jamaican children with and without ASD. Using univariable and multivariable GLMs, we investigated the additive and interactive effects of genotypes of the GST genes and ASD case status in blood arsenic concentrations. We did not find any statistically significant associations between the blood arsenic concentrations and the genotypes of *GSTT1* or *GSTM1* in Jamaican children with and without ASD. However, our findings revealed a significant interaction between ASD status and *GSTP1* genotype in relation to blood arsenic concentrations (*p* = 0.04 for the interaction terms based on the full genetic model). Specifically, based on the recessive genetic models, in TD Jamaican children we found that those with genotype Ile/Ile or Ile/Val for *GSTP1* had significantly higher geometric mean blood arsenic concentration than those with genotype Val/Val, (3.67 µg/L *vs.* 2.69 µg/L, *p* < 0.01). Although ASD cases with Ile/Ile or Ile/Val genotypes had adjusted geometric mean blood arsenic concentration of 3.71 µg/L compared to 3.29 µg/L for ASD cases with the Val/Val genotype, the effect of the *GSTP1* gene on adjusted geometric mean blood arsenic concentrations was not statistically significant for the ASD cases, (*p* = 0.29). However, the direction of the observed difference was consistent with that of the TD control group. The observed mean blood arsenic concentrations for TD Jamaican children were more than three times higher than 1 µg/L, which is considered to be the baseline for unexposed individuals in the US by the Agency for Toxic Substances and Disease Registry (ATSDR) [4]. We have previously reported that children with ASD in Jamaica have lower levels of seafood and vegetables consumption compared with TD Jamaican children, hence they may have lower exposure to arsenic through food they consume [22]. This may explain why we did not observe a significant difference in blood arsenic concentrations between individuals with different genotypes of *GSTP1* in the ASD cases.

Detoxification of arsenic is a complicated process that is currently not well understood [69]. It is believed that methylation of inorganic arsenic is involved in detoxification of this metalloid. However, methylated metabolites of arsenic, MMeAsIII and DMeAsIII, excreted in urine after exposure to inorganic arsenic are more toxic than inorganic arsenic itself [70]. Since we did not have data for urinary arsenic species, we were not able to use the primary or secondary methylation ratios to measure the individual methylation abilities of arsenic. However, we believe that the blood arsenic concentrations may serve as a surrogate biomarker representing how arsenic can be effectively metabolized and excreted, because total blood arsenic concentrations have been previously shown to be highly correlated with urinary arsenic concentration when adjusted for creatinine (Spearman r = 0.85) [62]. Our findings suggest that TD Jamaican children with the *GSTP1* Val/Val genotype have a significantly lower geometric mean blood arsenic concentration than those with other genotypes (*i.e.*, Ile/Ile or Ile/Val), perhaps due to a higher detoxification of arsenic from blood. To the best of our knowledge, we are the first to report such evidence supporting the possible role of *GSTP1* in arsenic regulation: A single amino acid substitution (Ile105Val) in *GSTP1* may produce a variant enzyme and seems to have a significant impact on blood arsenic concentration. Moreover, since differences in gene expression have been observed between children with ASD and TD children [71], and arsenic exposure has been associated with both hypo- and hyper-methylation at various genetic loci [72,73], it is possible that epigenetic alterations [74] at the *GSTP1* locus or elsewhere in the genome that were not assessed in our study may have also had an impact on blood arsenic concentrations. Further epidemiological studies and experimental studies using animal models may uncover the underlying mechanisms of this phenomenon.

It has been reported that the *GSTO* genes may be responsible for catalyzing the conjunction of cellular glutathione and MMAIII [27]. A study by Todorova *et al.* (2007) reported that genes that are similar to the GST genes in mammals play an important role in defense against reactive oxygen species and overall cellular detoxification metabolism related to arsenic in the yeast *S. cerevisiae* [75]. In the study by McCarty *et al.* (2007), a significant interaction was found between the secondary methylation ratio (DMA/MMA) and the *GSTT1* gene [76]. In addition, stratified analyses by *GSTT1* genotypes showed that (1) for individuals with the *GSTT1* wild type genotype, a 10-fold increase in the primary methylation (MMA/(AsIII+AsV)) ratio was associated with a 1.67 increased risk of skin lesions (95% CI: 1.06–2.64); and (2) for individuals with the *GSTT1* wild type, a 10-fold increase in secondary methylation ratio was associated with decreased odds of skin lesions compared to *GSTT1* null (OR = 0.87, 95% CI: 0.76–0.99). It was anticipated that perhaps *GSTT1* would have the same effect as *GSTO1*, or that *GSTT1* was in linkage disequilibrium with another polymorphism that is responsible for this observed effect. However, because we do not have data for urinary concentration and the detailed components of arsenic (e.g., organic *vs.* inorganic), our results cannot be directly compared with those from this study.

In a study by Agusa *et al.* (2010), it was shown that individuals with the heterozygous genotype of *GSTP1* Ile105Val have a higher capacity to metabolize inorganic arsenic (AsIII + AsV) to the methylated form (MMAV), but a lower metabolic capacity to metabolize AsV to AsIII [6]. Assuming that the methylated form, MMAV, is excreted and will not enter or stay in the blood, their findings are in agreement with ours.

Statistical power could be improved when identifying factors associated with blood arsenic concentrations by pooling data from ASD cases and TD controls and including an interaction term for ASD case status and *GSTP1* genotypes in the regression model. In this study we had 100 pairs of ASD cases and their age- and sex matched TD controls. In order to account for potential correlation between ASD cases and TD controls due to matching, we included 99 dummy variables that represented the 100 matched pairs in all GLMs. Since determination of potential confounding variables in models that involve interactive effects does not follow the classical definition of confounders in additive models, we adjusted for potential confounders that were determined *a priori* from our earlier report [22]. Potential confounding variables that were included in multivariable GLMs were SES (car ownership), maternal age at child’s birth, parental education levels, source of drinking water, consumption of “yam, sweet potato, or dasheen”, “carrot or pumpkin”, “callaloo, broccoli, or pak choi”, “cabbage”, “avocado”, and frequency of seafood consumption. Therefore, the adjusted effects reported for various genotypes of *GSTP1* have already taken into account for potential effects of various food consumed by the groups compared.

Previously, we have reported that blood arsenic concentrations in Jamaican children are about four times higher than those of children in the US or Canada [22]. We have also reported that factors such as sources of drinking water and consumption of fruits, vegetables, and seafood are associated with blood arsenic concentrations in Jamaican children. Our recent findings in this report indicate that about 77% of TD children in Jamaica (25% with genotype Ile/Ile and 52% with genotype or Ile/Val for *GSTP1*) may be at a higher risk of exposure to arsenic, hence at higher risk of aforementioned diseases or disorders that are associated with arsenic exposure. Since genetic risk factors are not modifiable, we recommend implementation of interventions focused on dietary and environmental factors that could help to reduce exposure to arsenic in Jamaican children as we have recommended in our previous report [22].

Previously, based on 65 matched pairs, we have also reported that blood arsenic concentrations in Jamaican children were not associated with ASD status [22]. Though not the main focus of this paper, we repeated the same analyses using the 100 matched pairs. Our findings from an additive multivariable GLM in this study are consistent with those that we reported earlier. However, in this study we found a significant interaction between ASD status and *GSTP1* in relation to blood arsenic concentrations in Jamaican children. This finding may suggest a more complex relationship between arsenic exposure and ASD in children. As shown in a twin study by Hallmayer *et al.* (2011), environmental factors may have a greater influence than genetic factors on the susceptibility to ASD. Characterization of environmental exposures, including arsenic, is therefore of importance to our understanding of the etiology of ASD [77]. The lack of association between blood arsenic concentrations and *GSTP1* genotypes for the ASD group requires further investigation.

## 5. Limitations

We acknowledge several limitations in this study. First, since the control children for this study were selected to match the ASD cases by sex and age from the Kingston area, they may not represent a random sample from the population of all children in Jamaica. Additionally, our controls belonged to a lower SES group than our ASD cases. Therefore, the findings reported in this study may not be generalizable to populations other than that in which the samples were selected. Due to limited resources, in this study we assessed only total blood arsenic concentrations. In addition, we acknowledge that we did not use fasting blood to measure the arsenic concentrations, which would probably provide a more consistent measurement of arsenic exposure. Moreover, we do not have the exact time of the day in which blood is drawn from children in this study. This limits our ability to control for the timing of blood draw as a potential confounding factor. However, we acknowledge that assessment of inorganic and organic urine arsenic concentrations would allow a better assessment for risk of exposure to inorganic arsenic in Jamaican children and a more detailed analysis of the role of GST genes in arsenic metabolism, as reported in McCarty *et al.* [76]. Although our analyses for food consumption (fruits, vegetables, and seafood) were conducted under the assumption that most products were grown and caught locally, we acknowledge that some participants may have consumed foods imported from other locations; however, we did not assess this possibility in the food frequency questionnaire. Also, it is possible that our analysis did not account for unmeasured confounding variables that may have a strong correlation with blood arsenic concentrations and the *GSTP1* genotypes in the ASD group. Finally, we acknowledge that it is possible that the observed association between the genotypes of *GSTP1* and the blood arsenic concentration may not necessarily represent an effect of *GSTP1*, but simply imply that *GSTP1* is on linkage disequilibrium with the true causal polymorphism that was not measured in this study.

## 6. Conclusions

In this article, we have shown that the TD children in Jamaica with *GSTP1* genotype Ile/Ile or Ile/Val had a significantly higher blood arsenic concentration than the TD children with genotype Val/Val. These findings are consistent with a possible role for *GSTP1* in the detoxification of arsenic. Although we observed a similar pattern for the ASD cases, this association was not statistically significant. Since higher levels of blood arsenic is associated with several diseases and disorders, our results also suggest that the effect of *GSTP1* genotype on the ability to metabolize arsenic may influence the risk of these conditions in TD children in Jamaica. Since genetic risk factors are not modifiable, we recommend implementation of interventions focused on dietary and environmental factors that could help to reduce exposure to arsenic in Jamaican children; as was recommended in our previous report.

## Supplementary Files

Supplementary File 1
