# Supplementary material for: Role of Metabolic Genes in Blood Arsenic Concentrations of Jamaican Children with and without Autism Spectrum Disorder
Source: Int J Environ Res Public Health. 2014 Aug 6;11(8):7874–95. doi: 10.3390/ijerph110807874 (PMC4143838; doi:10.3390/ijerph110807874)
Supplement: Supplementary File 1 [file ijerph-11-07874-s001.pdf]

Supplementary Material

Role of Metabolic Genes in Blood Arsenic Concentrations of Jamaican Children with and without Autism Spectrum Disorder

---

Detailed description of General Linear Models used to obtain the results reported in Table 3 (based on 100 matched pairs)

General Linear Models that assessed unadjusted effects of various exposure variables in blood arsenic concentrations of children with and without ASD can be represented by the following linear regression equations:

Models: $E(Y_i \middle| X_{1i}, X_{2i}, X_{12ij}) = \beta_0 + \beta_1 X_{1i} + \beta_{2k} X_{2ki} + \sum_{j=1}^{99} \beta_{3j} X_{3ij}$ *; k=1, 2,..., 27 that represent various exposure variables*

$$i = 1, 2, \ldots, 200 \ (number\ of\ children);\ j = 1, 2, \ldots, 99 (number\ of\ dummy\ variables\ representing\ the\ 100\ matched\ pairs);$$

$$Y_i = ln\ of\ blood\ arsenic\ concentrations\ of\ the\ i^{th}\ child;$$

$$X_{1i} = case\ status\ of\ the\ i^{th}\ child\ (ASD\ case = 1;\ TD\ control = 0);$$

$$X_{21i} = car\ ownership\ associated\ with\ family\ of\ the\ i^{th}\ child\ (\ No\ cars = 0; otherwise = 1);$$

$$X_{22i} = maternal\ age\ at\ child's\ birth\ associated\ with\ the\ i^{th}\ child\ (\text{"}maternal\ age \geq 35\ years\text{"} = 1;\ otherwise = 0);$$

$$X_{23i} = parental\ levels\ of\ education\ associated\ with\ the\ i^{th}\ child\ \ (both\ parents\ had\ up\ to\ high\ school = 0; otherwise = 1);$$

$$X_{24i} = source\ of\ drinking\ water\ associated\ with\ the\ i^{th}\ child\ (piped\ water = 1;\ otherwise = 0);$$

$$X_{25i} = consumption\ of\ \text{“}yam, sweet\ potato, or\ \ dasheen\text{”}\ associated\ with\ the\ i^{th}\ child\ (No\ consumption = 0;\ otherwise = 1);$$

$$X_{26i} = consumption\ of\ \text{“}carrot\ or\ pumpkin\text{”}\ associated\ with\ the\ i^{th}\ child\ (No\ consumption = 0;\ otherwise = 1);$$

$$X_{27i} = consumption\ of\ \ \text{“}lettuce\text{”}\ associated\ with\ the\ i^{th}\ child\ (No\ consumption = 0;\ otherwise = 1);$$

$$X_{28i} = consumption\ of\ \ \text{“}callaloo, broccoli, or\ \ pak\ choi\text{”}\ associated\ with\ the\ i^{th}\ child\ (No\ consumption = 0;\ otherwise = 1);$$

$$X_{29i} = consumption\ of\ \text{“}cabbage\text{”}\ associated\ with\ the\ i^{th}\ child\ (No\ consumption = 0;\ otherwise = 1);$$

$$X_{210i} = consumption\ of\ \text{“}string\ beans\text{”}\ associated\ with\ the\ i^{th}\ child\ (No\ consumption = 0;\ otherwise = 1);$$

$$X_{211i} = consumption\ of\ \text{“}tomatoes\text{”}\ associated\ with\ the\ i^{th}\ child\ (No\ consumption = 0;\ otherwise = 1);$$

$$X_{212i} = consumption\ of\ \text{“}ackee\text{”}\ associated\ with\ the\ i^{th}\ child\ (No\ consumption = 0;\ otherwise = 1);$$

$$X_{213i} = consumption\ of\ \text{“}avocado\text{”}\ associated\ with\ the\ i^{th}\ child\ (No\ consumption = 0;\ otherwise = 1);$$

$$X_{214i} = high\ seafood\ consumption\ associated\ with\ the\ i^{th}\ child\ (more\ than\ 6\ \ seafood\ meals\ per\ week = 1;\ otherwise = 0);$$

$$X_{215i} = frequency\ of\ seafood\ consumption\ associated\ with\ the\ i^{th}\ child = number\ of\ seafood\ meals\ consumed\ in\ a\ week;$$

$$X_{216i} = consumption\ of\ \text{“}salt\ water\ fish\text{”}\ associated\ with\ the\ i^{th}\ child\ (No\ consumption = 0;\ otherwise = 1);$$

$$X_{217i} = consumption\ of\ \text{“}fresh\ water\ fish\text{”}\ associated\ with\ the\ i^{th}\ child\ (No\ consumption = 0;\ otherwise = 1);$$

$$X_{218i} = consumption\ of\ \text{“}sardine, mackerel\text{”}\ associated\ with\ the\ i^{th}\ child\ (No\ consumption = 0;\ otherwise = 1);$$

$$X_{219i} = consumption\ of\ \text{“}tuna\text{”}\ associated\ with\ the\ i^{th}\ child\ (No\ consumption = 0;\ otherwise = 1);$$

$$X_{220i} = consumption\ of\ \text{“}salted\ fish\ \text{”}\ associated\ with\ the\ i^{th}\ child\ (No\ consumption = 0;\ otherwise = 1);$$

$$X_{221i} = consumption\ of\ \text{“}shellfish\text{”}\ associated\ with\ the\ i^{th}\ child\ (No\ consumption = 0;\ otherwise = 1);$$

$$X_{222i} = consumption\ of\ \text{“}shrimp\text{”}\ associated\ with\ the\ i^{th}\ child\ (No\ consumption = 0;\ otherwise = 1);$$

$$X_{223i} = GSTM1\ genotype\ associated\ with\ the\ i^{th}\ child\ (\text{"}DD\text{"} = 0; \text{"}I/I\text{"}\ or\ \text{"}I/D\text{"} = 1);$$

$$X_{224i} = GSTT1\ genotype\ associated\ with\ the\ i^{th}\ child\ (\text{"}DD\text{"} = 0; \text{"}I/I\text{"}\ or\ \text{"}I/D\text{"} = 1);$$

$$X_{225i} = \text{"}GSTP1(Ile/Ile)\ genotype\text{"}\ associated\ with\ the\ i^{th}\ child\ (GSTP1(Ile/Ile)\ genotype = 1;\ otherwise = 0);$$

$$X_{226i} = \text{“}GSTP1(Val/Val)\ genotype\text{”}\ associated\ with\ the\ i^{th}\ child\ (GSTP1(Val/Val)\ genotype = 1;\ otherwise = 0);$$

$$X_{227i} = \text{“}GSTP1(Ile/Val)\ genotype\text{”}\ associated\ with\ the\ i^{th}\ child\ (GSTP1(Ile/Val)\ genotype = 1;\ otherwise = 0);$$

$$X_{3ij} = \begin{cases} 1, if\ the\ i^{th}\ child\ in\ the\ j^{th}\ matched\ pair\ is\ an\ ASD\ case \\ 0, if\ the\ i^{th}\ child\ in\ the\ j^{th}\ matched\ pair\ is\ a\ TD\ control \end{cases};$$

$$E(Y_i | X_{1i}, X_{2ki}, \ldots,, X_{3ij})\ stands\ for\ mean\ ln\ blood\ arsenic\ concentrations\ given\ the\ X_{1i}, X_{2ki}, X_{3ij};$$

$$\beta_0, \beta_1, \beta_{2k}, \beta_{3j}\ are\ regression\ coefficients\ associated\ with\ the\ General\ Linear\ Models.$$

Detailed description of additive General Linear Models that are used to obtain the results reported in Table 4 (based on 65 matched pairs)

The univariable and final multivariable additive General Linear Models used that assessed possible association between blood arsenic concentrations and ASD status can be represented by the following linear regression equations:

For the univariable model: $E(Y_i \mid X_{1i}, X_{12ij}) = \beta_{10} + \beta_{11} X_{1i} + \sum_{j=1}^{64} \beta_{112j} X_{12ij}$;
For the final multivariable model: $E(Y_i \mid X_{1i}, X_{2i}, \ldots, X_{11i}, X_{12ij}) = \beta_{20} + \beta_{21} X_{1i} + \beta_{22} X_{2i} + \cdots + \beta_{211} X_{11i} + \sum_{j=1}^{64} \beta_{212j} X_{12ij}$;

$$i = 1, 2, \ldots, 130 \ (number\ of\ children);\ j = 1, 2, \ldots, 64 (number\ of\ dummy\ variables\ representing\ the\ 65\ matched\ pairs);$$
$$Y_i = ln\ of\ blood\ arsenic\ concentrations\ of\ the\ i^{th}\ child;$$
$$X_{1i} = case\ status\ of\ the\ i^{th}\ child\ (ASD\ case = 1;\ TD\ control = 0);$$
$$X_{2i} = car\ ownership\ associated\ with\ family\ of\ the\ i^{th}\ child\ (\ No\ cars = 0; otherwise = 1);$$
$$X_{3i} = maternal\ age\ at\ child's\ birth\ associated\ with\ the\ i^{th}\ child\ (\text{"}maternal\ age \geq 35\ years\text{"} = 1;\ otherwise = 0);$$
$$X_{4i} = Parental\ levels\ of\ education\ associated\ with\ the\ i^{th}\ child\ \ (both\ parents\ had\ up\ to\ high\ school = 0; otherwise = 1);$$
$$X_{5i} = source\ of\ drinking\ water\ associated\ with\ the\ i^{th}\ child\ (piped\ water = 1,\ otherwise = 0);$$
$$X_{6i} = consumption\ of\ \text{“}yam, sweet\ potato, or\ \ dasheen\text{”}\ associated\ with\ the\ i^{th}\ child\ (No\ consumption = 0;\ otherwise = 1);$$
$$X_{7i} = consumption\ of\ \text{“}carrot\ or\ pumpkin\text{”}\ associated\ with\ the\ i^{th}\ child\ (No\ consumption = 0;\ otherwise = 1);$$
$$X_{8i} = consumption\ of\ \ \text{“}callaloo, broccoli, or\ \ pak\ choi\text{”}\ associated\ with\ the\ i^{th}\ child\ (No\ consumption = 0;\ otherwise = 1);$$
$$X_{9i} = consumption\ of\ \text{“}cabbage\text{”}\ associated\ with\ the\ i^{th}\ child\ (No\ consumption = 0;\ otherwise = 1);$$
$$X_{10i} = consumption\ of\ \text{“}avocado\text{”}\ associated\ with\ the\ i^{th}\ child\ (No\ consumption = 0;\ otherwise = 1);$$
$$X_{11i} = frequency\ of\ seafood\ consumption\ associated\ with\ the\ i^{th}\ child = number\ of\ seafood\ meals\ consumed\ in\ a\ week;$$
$$X_{12ij} = \begin{cases} 1, if\ the\ i^{th}\ child\ in\ the\ j^{th}\ matched\ pair\ is\ an\ ASD\ case \\ 0, if\ the\ i^{th}\ child\ in\ the\ j^{th}\ matched\ pair\ is\ a\ TD\ control \end{cases} ;$$
$$E(Y_{1i} \mid X_{1i}, X_{12ij})\ stands\ for\ mean\ ln\ blood\ arsenic\ concentrations\ given\ X_{1i}, X_{12ij};$$
$$E(Y_{2i} \mid X_{1i}, X_{2i}, \ldots, X_{11i}, X_{12ij})\ stands\ for\ mean\ ln\ blood\ arsenic\ concentrations\ given\ X_{1i}, X_{2i}, \ldots, X_{11i}, X_{12ij};$$
$$\beta_{10}, \beta_{11}, \beta_{112j}\ are\ regression\ coefficients\ associated\ with\ the\ univaribale\ model;$$
$$\beta_{20}, \beta_{21}, \ldots, \beta_{211}, \beta_{212j}\ are\ regression\ coefficients\ associated\ with\ the\ final\ multivaribale\ model.$$

Detailed description of additive General Linear Models used to obtain the results reported in Table 4 (based on 100 matched pairs)

The univariable and final multivariable additive General Linear Models used that assessed possible association between blood arsenic concentrations and ASD status can be represented by the following linear regression equations:

For the univariable model: $E(Y_i \mid X_{1i}, X_{12ij}) = \beta_{10} + \beta_{11} X_{1i} + \sum_{j=1}^{99} \beta_{112j} X_{12ij}$;

For the final multivariable model: $E(Y_i \mid X_{1i}, X_{2i}, \ldots, X_{11i}, X_{12ij}) = \beta_{20} + \beta_{21} X_{1i} + \beta_{22} X_{2i} + \cdots + \beta_{211} X_{11i} + \sum_{j=1}^{99} \beta_{212j} X_{12ij}$;

$$i = 1, 2, \ldots, 200 \ (number\ of\ children);\ j = 1, 2, \ldots, 99 (number\ of\ dummy\ variables\ representing\ the\ 100\ matched\ pairs);$$

$$Y_i = ln\ of\ blood\ arsenic\ concentrations\ of\ the\ i^{th}\ child;$$

$$X_{1i} = case\ status\ of\ the\ i^{th}\ child\ (ASD\ case = 1;\ TD\ control = 0);$$

$$X_{2i} = car\ ownership\ associated\ with\ family\ of\ the\ i^{th}\ child\ (No\ cars = 0; otherwise = 1);$$

$$X_{3i} = maternal\ age\ at\ child\text{'}s\ birth\ associated\ with\ the\ i^{th}\ child\ (\text{"}maternal\ age \geq 35\ years\text{"} = 1;\ otherwise = 0);$$

$$X_{4i} = Parental\ levels\ of\ education\ associated\ with\ the\ i^{th}\ child\ (both\ parents\ had\ up\ to\ high\ school = 0; otherwise = 1);$$

$$X_{5i} = source\ of\ drinking\ water\ associated\ with\ the\ i^{th}\ child\ (piped\ water = 1,\ otherwise = 0);$$

$$X_{6i} = consumption\ of\ \text{“}yam, sweet\ potato, or\ dasheen\text{”}\ associated\ with\ the\ i^{th}\ child\ (No\ consumption = 0;\ otherwise = 1);$$

$$X_{7i} = consumption\ of\ \text{“}carrot\ or\ pumpkin\text{”}\ associated\ with\ the\ i^{th}\ child\ (No\ consumption = 0;\ otherwise = 1);$$

$$X_{8i} = consumption\ of\ \text{“}callaloo, broccoli, or\ pak\ choi\text{”}\ associated\ with\ the\ i^{th}\ child\ (No\ consumption = 0;\ otherwise = 1);$$

$$X_{9i} = consumption\ of\ \text{“}cabbage\text{”}\ associated\ with\ the\ i^{th}\ child\ (No\ consumption = 0;\ otherwise = 1);$$

$$X_{10i} = consumption\ of\ \text{“}avocado\text{”}\ associated\ with\ the\ i^{th}\ child\ (No\ consumption = 0;\ otherwise = 1);$$

$$X_{11i} = frequency\ of\ seafood\ consumption\ associated\ with\ the\ i^{th}\ child = number\ of\ seafood\ meals\ consumed\ in\ a\ week;$$

$$X_{12ij} = \begin{cases} 1, if\ the\ i^{th}\ child\ in\ the\ j^{th}\ matched\ pair\ is\ an\ ASD\ case \\ 0, if\ the\ i^{th}\ child\ in\ the\ j^{th}\ matched\ pair\ is\ a\ TD\ control \end{cases} ;$$

$$E(Y_{1i} \mid X_{1i}, X_{12ij})\ stands\ for\ mean\ ln\ of\ blood\ arsenic\ concentrations\ given\ X_{1i}, X_{12ij};$$

$$E(Y_{2i} \mid X_{1i}, X_{2i}, \ldots,, X_{12ij})\ stands\ for\ mean\ ln\ of\ blood\ arsenic\ concentrations\ given\ X_{1i}, X_{2i}, \ldots, X_{11i}, X_{12ij};$$

$$\beta_{10}, \beta_{11}, \beta_{112j}\ are\ regression\ coefficients\ associated\ with\ the\ univaribale\ model;$$

$$\beta_{20}, \beta_{21}, \ldots, \beta_{211}, \beta_{12j}\ are\ regression\ coefficients\ associated\ with\ the\ final\ multivaribale\ model.$$

Detailed description of General Linear Models used to obtain the results reported in Table 5 (based on 100 matched pairs)

General Linear Models that assessed unadjusted and adjusted effects of *GSTP1* in blood arsenic concentrations of children with and without ASD can be represented by the following linear regression equations:

For the unadjusted model: $E(Y_i | X_{1i}, X_{2i}, X_{12ij}) = \beta_{10} + \beta_{11} X_{1i} + \beta_{12} X_{2i} + \beta_{13} X_{1i} * X_{2i} + \sum_{j=1}^{99} \beta_{113j} X_{13ij}$;

For the final adjusted model: $E(Y_i | X_{1i}, X_{2i}, \ldots, X_{11i}, X_{12ij}) = \beta_{20} + \beta_{21} X_{1i} + \beta_{22} X_{2i} + \cdots + \beta_{212} X_{12i} + \beta_{13} X_{1i} * X_{2i} + \sum_{j=1}^{99} \beta_{213j} X_{13ij}$;

$$i = 1, 2, \ldots, 200\ (number\ of\ children);\ j = 1, 2, \ldots, 99 (number\ of\ dummy\ variables\ representing\ the\ 99\ matched\ pairs);$$

$$Y_i = ln\ of\ blood\ arsenic\ concentrations\ of\ the\ i^{th}\ child;$$

$$X_{1i} = case\ status\ of\ the\ i^{th}\ child\ (ASD\ case\ = 1;\ TD\ control = 0);$$

$$X_{2i} = GSTP1\ genotype\ associated\ with\ the\ i^{th}\ child\ (GSTP1\ is\ categorized\ into\ 2\ levels);$$

$$X_{3i} = car\ ownership\ associated\ with\ family\ of\ the\ i^{th}\ child\ (\ No\ cars = 0; otherwise = 1);$$

$$X_{4i} = maternal\ age\ at\ child's\ birth\ associated\ with\ the\ i^{th}\ child\ (\text{"}maternal\ age \geq 35\ years\text{"} = 1;\ otherwise\ = 0);$$

$$X_{5i} = Parental\ levels\ of\ education\ associated\ with\ the\ i^{th}\ child\ \ (both\ parent\ up\ to\ high\ schhol\ = 0; otherwise = 1);$$

$$X_{6i} = source\ of\ drinking\ water\ associated\ with\ the\ i^{th}\ child\ (piped\ water = 1,\ otherwise = 0);$$

$$X_{7i} = consumption\ of\ \text{``}yam, sweet\ potato, or\ \ dasheen\text{''}\ associated\ with\ the\ i^{th}\ child\ (No\ consumption = 0;\ otherwise = 1);$$

$$X_{8i} = consumption\ of\ \text{``}carrot\ or\ pumpkin\text{''}\ associated\ with\ the\ i^{th}\ child\ (No\ consumption = 0;\ otherwise = 1);$$

$$X_{9i} = consumption\ of\ \ \text{``}callaloo, broccoli, or\ \ pak\ choi\text{''}\ associated\ with\ the\ i^{th}\ child\ (No\ consumption = 0;\ otherwise = 1;$$

$$X_{10i} = consumption\ of\ \text{``}cabbage\text{''}\ associated\ with\ the\ i^{th}\ child\ (No\ consumption = 0;\ otherwise = 1);$$

$$X_{11i} = consumption\ of\ \text{``}avocado\text{''}\ associated\ with\ the\ i^{th}\ child\ (No\ consumption = 0;\ otherwise = 1);$$

$$X_{12i} = frequency\ of\ seafood\ consumption\ associated\ with\ the\ i^{th}\ child = number\ of\ seafood\ meals\ consumed\ in\ a\ week;$$

$$X_{13ij} = \begin{cases} 1, if\ the\ i^{th}\ child\ in\ the\ j^{th}\ matched\ pair\ is\ an\ ASD\ case \\ 0, if\ the\ i^{th}\ child\ in\ the\ j^{th}\ matched\ pair\ is\ a\ control \end{cases};$$

$$E(Y_{1i}|\, X_{1i}, X_{13ij})\ stands\ for\ mean\ ln\ of\ blood\ arsenic\ concentrations\ given\ X_{1i}, X_{13ij};$$
$$E(Y_{2i}|X_{1i}, X_{2i}, \ldots, , X_{13ij})\ stands\ for\ mean\ ln\ of\ blood\ arsenic\ concentrations\ given\ the\ X_{1i}, X_{2i}, \ldots, X_{12i}, X_{13ij};$$
$$\beta_{10}, \beta_{11}, \beta_{12}, \beta_{113j}\ are\ regression\ coefficients\ associated\ with\ the\ univaribale\ model;$$
$$\beta_{20}, \beta_{21}, \ldots, \beta_{212}, \beta_{213j}\ are\ regression\ coefficients\ associated\ with\ the\ final\ multivaribale\ model.$$

© 2014 by the authors; licensee MDPI, Basel, Switzerland. This article is an open access article distributed under the terms and conditions of the Creative Commons Attribution license (http://creativecommons.org/licenses/by/3.0/).
